# Negative Mode I Loading During Buckling-Induced Delamination and Its Impact

**DOI:** 10.1007/s11837-026-08570-5

**Published:** 2026-08-03

**Authors:** Stanislav Zak, Hannah Arnfelser, Neville R. Moody, Megan J. Cordill

**Affiliations:** 1 Erich Schmid Institute for Materials Science, Austrian Academy of Sciences, Jahnstraße 12, 8700 Leoben, Austria; 2 Sandia National Labororatories, Livermore, CA 94551-0969 USA; 3 Lehrstuhl für Materialphysik, Montanuniversität Leoben, Jahnstraße 12, 8700 Leoben, Austria

## Abstract

In modern engineering, combinations of substrate structures and thin coatings is ever present. Be it in protective applications, microelectronics, or with micro-electromechanical systems, the adhesion between the substrate material and thin film is crucial, and delamination is often the reason for the failure of the components. Therefore, the thin-film adhesion measurement is extremely important for the longevity and reliability of micro-scale components. One of the available methods is the buckling-induced delamination technique, using the compressive residual stresses in thin films to cause the films to delaminate and form a buckle. This method is well established within the material science community; however, one misconception still persists, that of discarding the negative mode I loading at the delamination crack front. The practice has led to assuming some buckling to be under pure mode II loading, but new analysis shows that the negative mode I loading pushes crack surfaces towards each other, increasing friction and apparent adhesion energy. This work presents the first description of the negative mode I influence in the case of buckling-induced delamination and the possibilities it could open for more investigation.

## Introduction

The usage of thin films and coatings dates back as far as 5000 BC^1^ for aesthetics and a crude protection against the elements or day-to-day use on tools. However, the start of the research in terms of material science connected to thin films and coatings dates back only several decades.^1–4^ Regardless of their use and loading, the adhesion between a coating or thin film and the substrate has to be kept as high as possible, since no-matter the actual strength of individual materials in the thin film–substrate system, the interface between two dissimilar materials is considered a weak link, and the weakness is present more when largely dissimilar materials are combined. Therefore, the measurement of interface fracture energy between the thin film and the substrate is very important for modern technologies.

**Fig. 1:**
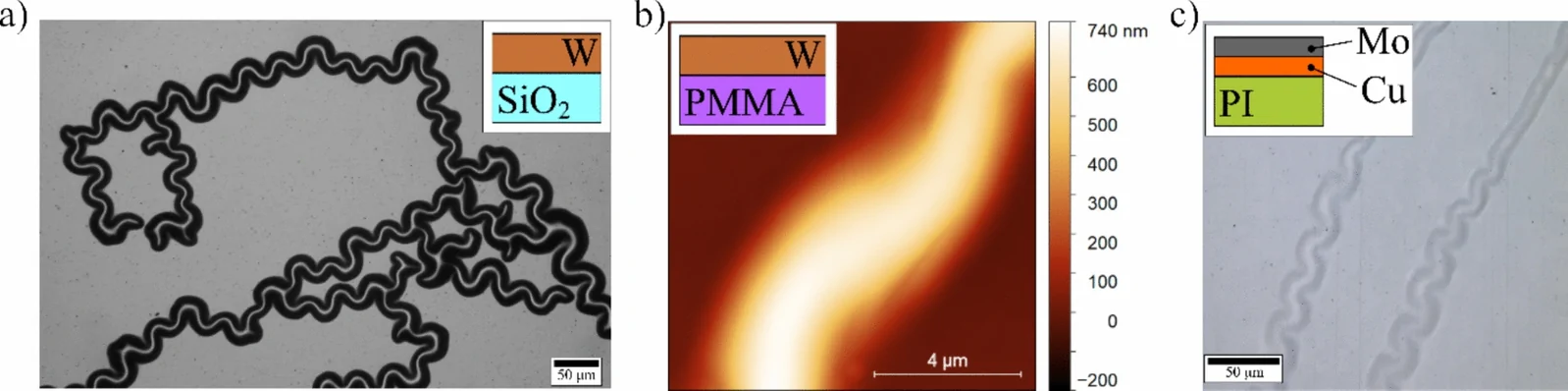
Buckles observed on each of the material systems: (a) W-SiO_2_ (telephone-cord buckle), optical microscope view, obtained from raw data from Cordill et al.,^21^ (b) W-PMMA (straight buckle), height image, obtained from raw data from Moody et al.,^37^ (c) Mo-Cu-PI (mixed buckle), optical microscope view.

There are many ways to measure the thin-film adhesion energy, depending on the loading and quantitative or qualitative aim of the experiment. Experimentally straightforward methods, with results depending strongly on the quality of the setup, are scratch, peel, and pull tests.^5^ Scaling down these methods into micro-scale thin films only magnifies the error caused by the need for perfect alignment. In the region of smaller scales, nanoindentation-based techniques are usually employed (e.g., cross-sectional nanoindentation^6,7^ or top-down nanoindentation initiating the delamination^8–12^). Another option is to employ the residual stresses in the thin film after deposition. This method, which originated in works by Hutchinson and Suo^13,14^ from the early 1990s, uses the compressive residual stress, *σ*, in the film and its tendency to deform the film, resulting in delaminated buckles in a form of telephone-cord or straight shapes, such as those clearly visible on the example images of buckles in Fig. 1. This method, called spontaneous buckling-induced delamination, has obvious advantages over nanoindentation techniques, with much less sensitivity to the experimental setup and no need for additional instrumentation, such as a nanoindenter. Additionally, a large quantity of buckles can be measured on one sample, leading to statistically significant datasets. Since this method is the main focus of the present manuscript, its explanation and execution are described in more detail in the “Materials and Methods” section.

Besides the original development of the method by Hutchinson and Suo,^13,14^ the practical application of this method was extended by Gerberich, inducing the buckling through nanoindentation,^9,11,15–17^ thin-film compressive stress,^18–22^ or with the adaptation of the method to compression-induced buckling.^23–26^ These and other works (e.g., Ref. 27–31) show the wide range of usability of the buckling-induced delamination method and its versatility to being modified for special cases or for slightly different loading regimes.

**Fig. 2:**
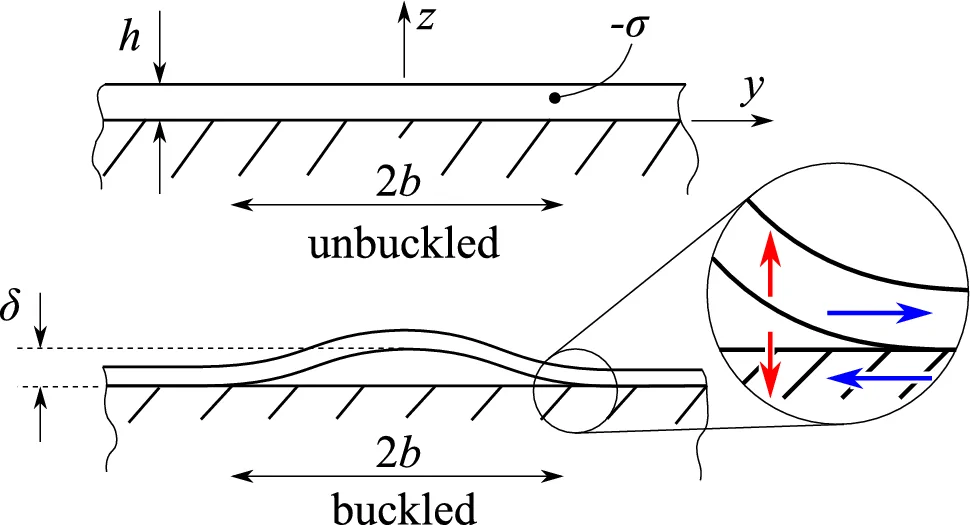
Schematic of the buckle geometry and behavior with the Hutchinson and Suo model^14^ (re-drawn according to the original image from Hutchinson and Suo^14^ with mode-mixity addition).

However, the buckling-induced delamination method has some simplifications and limitations which have remained unchanged since its development by Hutchinson and Suo.^14^ The base assumption that both the substrate and the thin-film deforming purely elastically is considered to be mostly achievable, while the case of plastic deformation of the underlying films,^32^ substrate,^33^ or within the buckle itself^34,35^ has been previously discussed. The second assumption regarding buckling-induced delamination lies within the elastic mismatch between the film and the substrate. In the original work, the elastic mismatch influence was described only for a limited number of materials;^13,14^ however, a more general approach with the use of numerical modeling showed the significant influence of the elastic mismatch as well as the improved solution.^30,36^ Within the base method, there is one additional assumption, which has so far not been investigated or challenged. This last assumption is connected to the mode mixity at the delamination crack front, schematically depicted by the red and blue arrows in the detail in Fig. 2 (red, vertical arrows represent normal mode I loading, and blue, horizontal arrows represent shear mode II loading). The ratio of these two modes (mode II to mode I) can be recalculated through a tangent function into a mode-mixity angle Ψ and is specific for each buckle. If the mode-mixity angle equals 0°, the case of pure mode I loading is present, while, if the mode-mixity angle equals ± 90°, the buckle is loaded in pure shear mode II. Any combination of mode I and II loading would be described by an angle not equal to 0° or ± 90°. Previously, it was assumed that, for buckling-induced delamination, only negative values of Ψ are valid, and that if the model yields a positive value of Ψ, it has to be considered as − 90°, without any special explanation,^14^ technically truncating the whole half-space (positive Ψ values) of the results.

With the assumption of ignoring the positive mode-mixity values, its origins and usefulness are the topic of the current paper. The reasons of original thought behind not depreciating the positive mode-mixity into a pure mode II loading is discussed alongside the experimental results, showing that the truncation approach is outdated. Proper use of positive Ψ values can actually lead to a better explanation of the whole buckling-delamination process, while adding a completely new way of buckle-delamination analysis with new assumptions and the inclusion of contact pressure between the crack faces.

## Materials and Methods

### Thin Film Material Systems

The works by the research group associated with Gerberich have presented results on many different material systems. Original measurements were available to the authors of this study. From the available datasets, two previously published material systems exhibit seemingly pure mode II loading (i.e., mode-mixity angle equals − 90°). More careful look on the actual measurements shows the actual neglecting of mode I component according to the widely applied consensus that, if Ψ > 0°, then Ψ = − 90°. These systems are thin W film on two substrates, brittle SiO_2_ summarized in Cordill et al.^21^ (with example microscope image of a telephone-cord buckle in Fig. 1a) and on relatively soft polymethyl methacrylate (PMMA) by Moody et al.^37,38^ (with example height image of a mostly straight buckle in Fig. 1b). In addition, a newly-measured Mo-Cu bi-layer deposited on a compliant polyimide (PI) substrate is included (with example microscope image of a mixed buckle in Fig. 1c).

The material systems for which the results were obtained from the literature are briefly described according to a summary of the deposition and measurement methods mentioned in the original manuscripts. For more clarifications, the readers are referred to the respective sources.

#### W-SiO_2_

Thin W film was deposited with various thicknesses, ranging from 100 nm to 300 nm. All the samples were sputter-deposited using DC magnetron sputtering in argon with chamber pressures between 2.9 and 3.9 mTorr and a power of 55 W, resulting in compressive residual stresses between 2 GPa and 4 GPa. Regions of the film spontaneously delaminated in the common telephone cord buckle morphology (Fig. 1a) and in the less common telephone cord-to-straight buckle morphology. The material properties used for adhesion energy calculations were: for the W thin film, the elastic modulus was assumed as *E*_W_ = 410 GPa and its Poisson’s ratio as *υ*_W_ = 0.28,^39,40^ while, for the SiO_2_ substrate, the elastic modulus was *E*_SiO2_ = 70 GPa and its Poisson’s ratio *υ*_SiO2_ = 0.17.^41,42^

#### W-PMMA

The W films on PMMA substrates were sputter-deposited to thicknesses of 100–400 nm with a residual compressive stress of 1.7 GPa. Straight buckles (with a minor degree of waviness) formed spontaneously on the PMMA substrates following film deposition (Fig. 1b). Through-substrate optical observations revealed matching buckle patterns along the film–substrate interface, indicating that delamination occurred for large and small buckles. For the W thin film, the same material properties as for the W-SiO_2_ material system were used (see “W-SiO_2_” section). The PMMA was analytically modeled with a Young’s modulus of *E*_PMMA_ = 2.94 GPa and Poisson’s ratio of *υ*_PMMA_ = 0.375.^43–45^

#### Mo-Cu-PI

A bi-layer consisting 100 nm of Mo on top of 100 nm of Cu, supported by a 50-*μ*m-thick PI substrate (Upilex-S) represents the newly-measured material system. Both components of the bi-layer were deposited using an industrial-scale magnetron sputter system (FHR Line 600 V) equipped with a planar Cu target (600 × 125 mm, 99.9995% purity; FHR Anlagenbau, Ottendorf-Okrill, Germany) and a rotary Mo target (Ø 125 × 600 mm, 99.97% purity; Plansee, Reute, Austria). Prior to deposition, the PI was pre-cut to 70 × 9 mm strips using a Cricut Explore Air 2, and ultrasonically cleaned in an ethanol bath for 5 min. After mounting in the deposition chamber, the substrates were plasma-etched for 380 s at 0.8 kW under 0.34 Pa (180 sccm Ar flow rate). The base pressure in the deposition chamber was 2 × 10^−6^ Pa. The bi-layer was deposited with an Ar flow rate of 300 sccm, corresponding to a pressure of 0.52 Pa. Direct-current powers of 1.5 kW for the Cu and 3.5 kW for the Mo target were used. The Cu layer deposition was in a dynamic mode and consisted of 5 cycles, and the deposition Mo layer was in a static mode with the time of 32 s. During deposition, the PI substrates were held at ground potential, without additional external heating. Areas of the film spontaneously delaminated in a mix of telephone cord and straight buckles (Fig. 1c). For the material properties, the Mo layer was represented with a Young’s modulus of *E*_Mo_ = 329 GPa and its Poisson’s ratio of *υ*_Mo_ = 0.29,^46–48^ Cu thin film with a Young’s modulus of *E*_Cu_ = 117 GPa and its Poisson’s ratio of *υ*_Cu_ = 0.34,^49–51^ and the PI substrate with a Young’s modulus of *E*_PI_ = 3.6 GPa and its Poisson’s ratio of *υ*_PI_ = 0.34.^52–54^

### Buckling-Induced Thin-Film Delamination

The spontaneous buckling-induced thinfilm delamination method by Hutchinson and Suo^14^ was applied on thin films deposited with compressive residual stresses high enough to initiate debonding along the interface between the thin film and the substrate or on bi-layers where the actual thin film material of interest cannot be deposited with compressive residual stress; instead, a second over-layer is deposited, pulling the thin bi-layer from the substrate by its own compressive stress, similar to the Mo-Cu-PI system.

If the compressive residual stress is high enough (generally in the GPa range), the thin film is pushed upwards and results in a delamination buckle with a width 2*b* and height *δ* (see Fig. 2). From the buckle dimensions, the thin-film elastic modulus *E*_1_, Poisson’s ratio *υ*_1_, and its thickness *h*, the adhesion energy of the thin film Γ can be calculated as Ref. 14:

1 $$ {\Gamma }\left( {\Psi } \right) = \frac{{\left( {1 - \vartheta_{1}^{2} } \right){\mathrm{h}}}}{{2{\mathrm{E}}_{1} }}\left( {{\upsigma } - {\upsigma}_{{\mathrm{c}}} } \right) \cdot \left( {{\upsigma } + 3{\upsigma}_{{\mathrm{c}}} } \right), $$

where the critical buckling stress* σ*_c_, can be calculated from the buckle dimensions:

2 $$ \sigma_{{\mathrm{c}}} = \frac{{\pi^{2} }}{12}\frac{{E_{1} }}{{\left( {1 - \vartheta_{1}^{2} } \right)}}\left( \frac{h}{b} \right)^{2} . $$

The residual stress, *σ*, in the thin film can either be measured (e.g., by X-ray diffraction^35^) or calculated from the relationship between buckle height and critical stress from Eq. 2:

3 $$ \delta = \left[ {\frac{4}{3}\left( {\frac{\sigma }{{\sigma_{{\mathrm{c}}} }} - 1} \right)} \right]^{\frac{1}{2}} . $$

In case of bi-layers deposited on substrate, the model has to be modified to accommodate for the uneven stiffness of the bi-layer using the moment of inertia, *I*_T_, of the system:^10^

4 $$ I_{{\mathrm{T}}} = \mathop \sum \limits_{i = 1}^{2} \frac{1}{12}n_{{\mathrm{i}}} kh_{{\mathrm{i}}}^{3} + n_{{\mathrm{i}}} kh_{{\mathrm{i}}} \left( {\overline{Y} - y_{{\mathrm{i}}} } \right)^{2} , $$

where $\overline{Y}$ is the composite centroid, considering the different moduli, *E*_i_, of the films, *y*_i_ is the centroid of each layer, and *h*_i_ is the thickness of each individual layer. In Eq. 4, *n* is necessary to account for the disparate Young’s moduli of the delaminated films. The variable *k* will cancel out once the critical buckling stress, *σ*_c bi-layer_, is computed:

5 $$ \sigma_{{\text{c, bi - layer}}} = \frac{{\pi^{2} }}{{khb^{2} }}\left( {\frac{{E_{1} }}{{1 - \vartheta_{1}^{2} }}} \right) \cdot I_{{\mathrm{T}}} . $$

For the bi-layers, the evaluation of the residual stress, *σ*, and the adhesion energy, Γ, follows Eqs. 1 and 3, only Eq. 2 being modified. For more descriptions and the origin of this bi-layer modification, the reader is referred to the original work, Kreise et al.^10^

Since the geometry of the delaminated buckle and the residual stress loading does not allow for pure mode I normal loading, the spontaneous buckling-induced delamination will be always a mixed-mode I + II case (i.e., a combination of normal mode I loading and in-plane shear mode II loading, usually described by the stress intensity factors, *K*_I_ and *K*_II_, respectively). A schematic of the loading is presented in Fig. 2. This combination of loading modes is described through the mode-mixity angle, Ψ, and the evaluated adhesion energy should be always represented as a function of this parameter:^14^

6 $$ \tan \left( \Psi \right) = \frac{{K_{{{\mathrm{II}}}} }}{{K_{{\mathrm{I}}} }} = \frac{{4\cos \left( \omega \right) + \sqrt 3 \frac{\delta }{h}\sin \left( \omega \right)}}{{ - 4\sin \left( \omega \right) + \sqrt 3 \frac{\delta }{h}\cos \left( \omega \right)}}. $$

where the variable, *ω*, is related to the elastic mismatch between the thin film and the substrate, which was investigated and described in previous works.^30,36^

In general, the mode-mixity angle values can be between − 90° and 90°, with 0° meaning pure mode I loading and ± 90° meaning pure mode II loading. However, for buckling-induced delamination, it was until now assumed that only negative values of Ψ can occur, and that, if the analytical model reaches positive values, it has to be assumed to be pure shear mode II, i.e., Ψ = − 90°. In the following, this original assumption as well as a broader approach allowing for positive Ψ values will be compared and discussed.

### Buckle Height Imaging

For the measurement of the buckle dimensions (width 2*b* and height *h*), two different methods were used in regard to the sample types. The newly created samples (Mo-Cu-PI) were imaged with the confocal laser scanning microscope (LEXT OLS 4100; Olympus), using a violet (405-nm) laser, whereas the full height image and profiles over the buckles in multiple locations were captured, resulting in 251 measured buckle profiles over straight and telephone-cord buckles. The samples from Cordill et al.^21^ and Moody et al.^37,38^ were measured with contact mode atomic force microscopy with a Digital Instruments Nanoscope IIIa, and at least 30 measurements per sample were taken across the observed buckles.

## Results

As described in the “Materials and Methods” section, the experimental work was performed on three material systems with two being obtained as raw data from the literature. Therefore, the results are also separated according to the material systems, even though the observed effects are similar. These will be discussed together in the “Discussion” section.

### W-SiO_2_ Material System

The results of the W-SiO_2_ material system were obtained from Cordill et al.^21^ However, in the published manuscript, only the mean values of different measurements are available. For a more comprehensive study of the results, we analyzed the raw buckle measurements. Therefore, each individual data point could be investigated.

Before the actual analysis of the positive values of mode-mixity angle Ψ, one persistent issue had to be addressed, the effect of elastic mismatch between the thin film and the substrate through the phase factor *ω* (see Eq. 6), which was, in the original work, assumed to be 52.1°, thus for no mismatch between the materials. In reality, the elastic mismatch between W and SiO_2_ can be described by the Dundurs parameters^55,56^ with *α* = 0.721 and *β* = 0.299. Using the relationship in Žák et al.,^30,36^ this elastic mismatch influences the mode mixity through phase factor *ω* = 50.6°. Therefore, the presented mode-mixity angles for the W-SiO_2_ material system were re-calculated from the raw data using this phase factor value.

The usage of the modified phase factor leads to all of the mode-mixity angles from this dataset to be positive, similar to the original work. However, the focus here is on the implications of allowing the positive mode mixity. From the newly evaluated results, the dataset splits into two subsets.

**Fig. 3:**
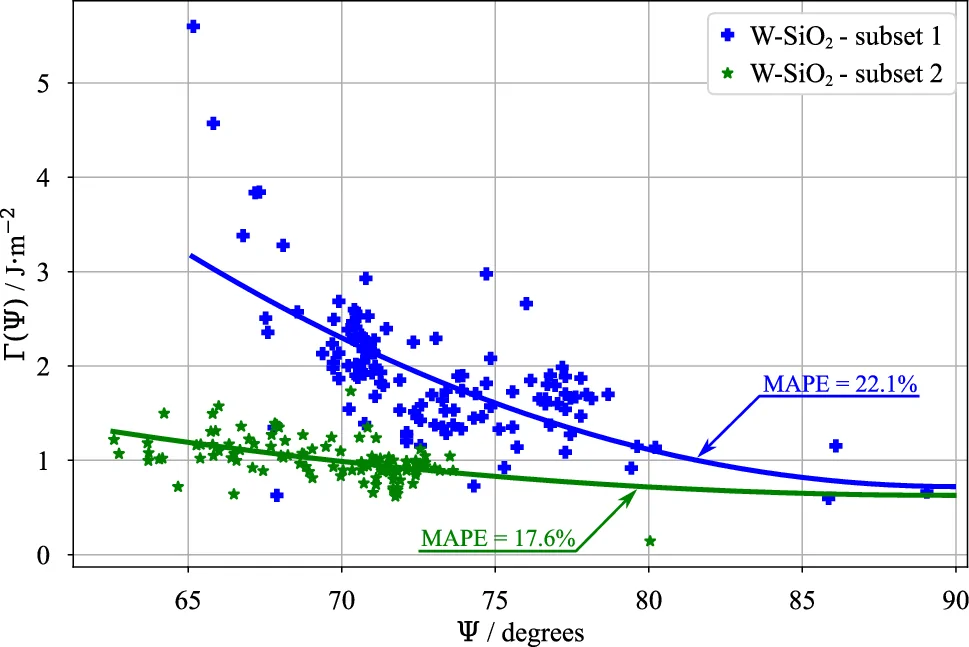
Re-calculated results from Cordill et al.^21^ using the phase factor value of 50.6° and allowing the occurrence of a positive mode-mixity angle.

As is clearly visible in Fig. 3, the values of Γ(Ψ) are not constant, as the original theory about only pure mode II loading suggests. There is a distinct trend and the results split into two subsets, which was not observable before. The two subsets differ in the film thicknesses, *h*, and in the calculated residual stresses, *σ*, with no directly observable correlation, dividing the measurements into two separate groups at the film thickness or stress levels. The data points presented in Fig. 3 could also be fitted with a quadratic function (constrained to have a minimum at 90°, see the solid lines in Fig. 3), with the relatively good quality of the fits represented by the mean absolute percentage error (MAPE). Extrapolating the fitted curves towards pure mode II loading at 90° leads to Γ_II, W-SiO2-01_ = 0.72 ± 0.12 J m^−2^ and Γ_II, W-SiO2-02_ = 0.62 ± 0.06 J m^−2^ for subsets 1 and 2, respectively. This is still within the range of what was reported in the original work; however, both slightly lower and with smaller standard deviations.

### W-PMMA Material System

The results on the W thin film deposited on the PMMA substrate were obtained from the original buckle measurements related to works by Moody et al.^37,38^ Similar to the W-SiO_2_ material system, the original, raw buckle measurements were adapted according to real elastic mismatch between the substrate and the thin film (*α* = 0.985 and *β* = 0.196), with the respective value of the phase factor *ω* = 71.5°, using the relationship in Žák et al.^30,36^

The results, after including the true effects of the elastic mismatch, shifted in comparison to the original analysis, not only presenting the positive values of mode-mixity angle Ψ but also some “standard” behavior with negative values of Ψ. Therefore, both sides of the < − 90°, 90° > interval have to be investigated.

**Fig. 4:**
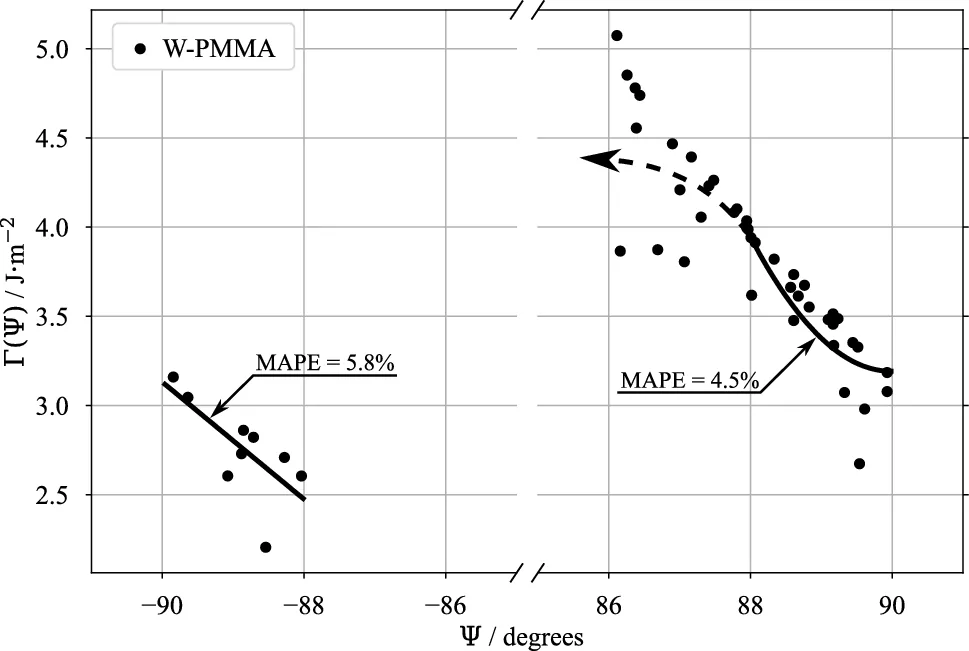
Re-calculated results from Moody et al.^37,38^ using the phase factor value of 71.5° and allowing for the occurrence of positive mode-mixity angles.

While smaller amounts of data for the W-PMMA material system were available (in comparison to the W-SiO_2_ system), Fig. 4 shows similar trends to Fig. 3, as well as the behavior known for buckling delamination. At first, the negative part of the graph shows a rapid decrease of Γ(Ψ) values with increasing mode-mixity angle (i.e., the introduction of mode I loading to pure mode II), which is an expected behavior (the pure mode II value for Ψ = − 90° should always be higher than the pure mode I value for Ψ = 0°).^14,57–59^ Fitting the negative part of the graph with a linear function (the number of data points does not allow a better function fit, although the linear fit results in a very good value of MAPE) leads to a pure mode II adhesion energy for Ψ = − 90° of Γ_II, W-PMMA_(− 90°) = 3.12 ± 0.16 J m^−2^. This value is consistent with the original work,^37,38^ albeit giving better standard deviations and a bit more confidence in the results, showing the actual data point progression.

On the other hand, the results which exhibit positive mode-mixity angles, while similar to those in Fig. 3, exhibit different behavior than ones with negative mode-mixity angles. The value of Γ(Ψ) is decreasing with increasing Ψ. This part of the data can be fitted with a similar quadratic function as in Fig. 3, with a very low value of MAPE; however, the fit accuracy is reduced for Ψ < 87.5°. For lower values of Ψ, fitting suggests the formation of plateau, which will be further discussed in the “Discussion” section. Nevertheless, the quadratic fit results in pure mode II adhesion energy for positive mode mixity as Γ_II, W-PMMA_(90°) = 3.19 ± 0.05 J m^−2^. This value is almost identical to that obtained from the fit towards Ψ = − 90°.

### Mo-Cu-PI Material System

The results on Mo-Cu-PI material system have not been previously published in their entirety and, as presented, are original measurements performed within this work. However, some of the data points were already introduced in Zak et al.^30^ The analysis was performed using Eq. 1 together with Eqs. 3, 4, 5, and 6, since this is the case of a bi-layer. The elastic mismatch from the composite bi-layer and the substrate is described with *α* = 0.948 and *β* = 0.229, resulting in a phase factor *ω* = 66.8°,^30,36^ leading to both positive and negative mode-mixity angles.

**Fig. 5:**
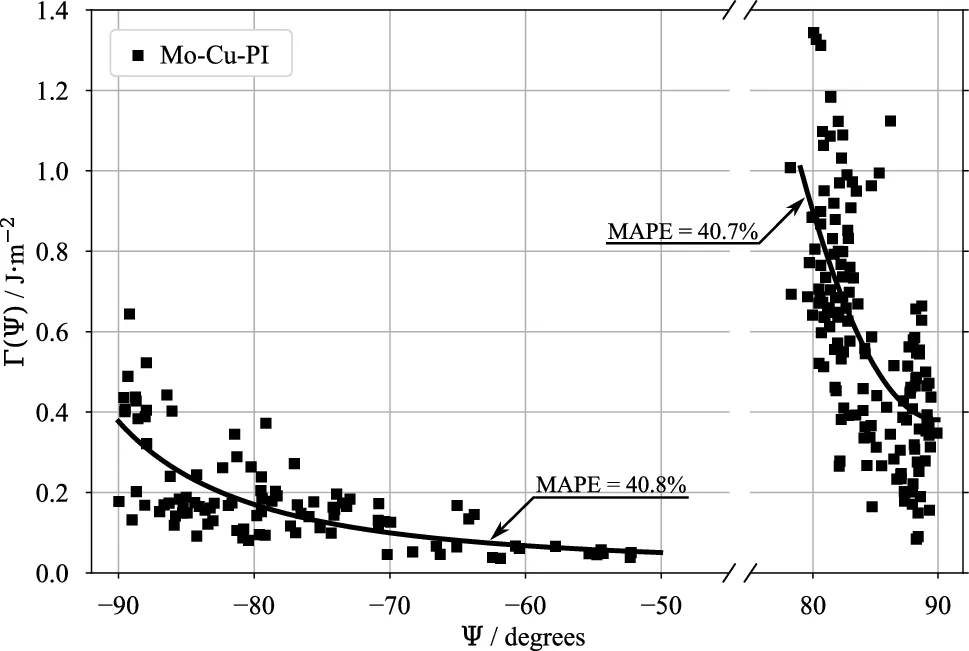
Newly obtained results from buckles measured on the Mo-Cu-PI bi-layered system.

The negative Ψ part of Fig. 5 follows the expected behavior and can be fitted with a function relating to the decay of adhesion energy from pure mode II to pure mode I:^14,59^

7 $$ {\Gamma }\left( {\Psi } \right) = {\Gamma}_{{\mathrm{I}}} \left[ {1 + {\mathrm{tan}}^{2} \left\{ {\left( {1 - \lambda } \right){\Psi }} \right\}} \right], $$

where Γ_I_ is the adhesion energy for pure mode I loading (when Ψ = 0°), comparable with critical fracture energy, *G*_I,c_, and *λ* is a parameter describing the mode II involvement in the fracture process; in this case, both were set as unconstrained fitting parameters. Since the experimental data are somewhat scattered, the evaluated MAPE of the fit is rather large. However, the experimental data follow the model well and behave as expected.

Fitting of Eq. 7 in the negative part of Fig 5 yields the value of mode I adhesion Γ_I, Mo-Cu-PI_ = 0.029 ± 0.005 J m^−2^ and the value of pure mode II of Γ_II, Mo-Cu-PI_(− 90°) = 0.376 ± 0.026 J m^−2^. The measured values are low, but such a low adhesion of metals on untreated, smooth polymeric substrates has already been reported before e.g. for the the Mo-PI system with the buckling–delamination method^30^ or for the Cu-PI system, measured via peel testing,^60–62^ and is, therefore, expected.

The results for the positive mode-mixity angle show similar behavior to the other presented cases with a rapid decrease of the Γ(Ψ) value with increasing angle Ψ above 80°. Similarly to the previous material systems, the data points with positive mode-mixity angles could be fitted with a quadratic function leading to Γ_II, Mo-Cu-PI_(90°) = 0.381 ± 0.03 J m^−2^. This value corresponds very well with the one obtained for Ψ = − 90°. While the fit with the quadratic function seems to work well with this dataset, it has to be noted that the fitted MAPE value is considerably larger than those from the W-SiO_2_ and W-PMMA cases. This is most probably caused by the large scatter of the experimental results over a small Ψ angle range, and to possible differences between single- and double-layer thin-film systems (which is not the focus of this manuscript). Nevertheless, the quadratic function fit follows the same progression and is deemed appropriate to show the correlation between mode II Γ_II_ values for Ψ =  ± 90°.

## Discussion

To be able to properly discuss the implications of positive mode-mixity, it has to be first understood what the positive or negative mode-mixity angles in buckling–delamination mean.

**Fig. 6:**
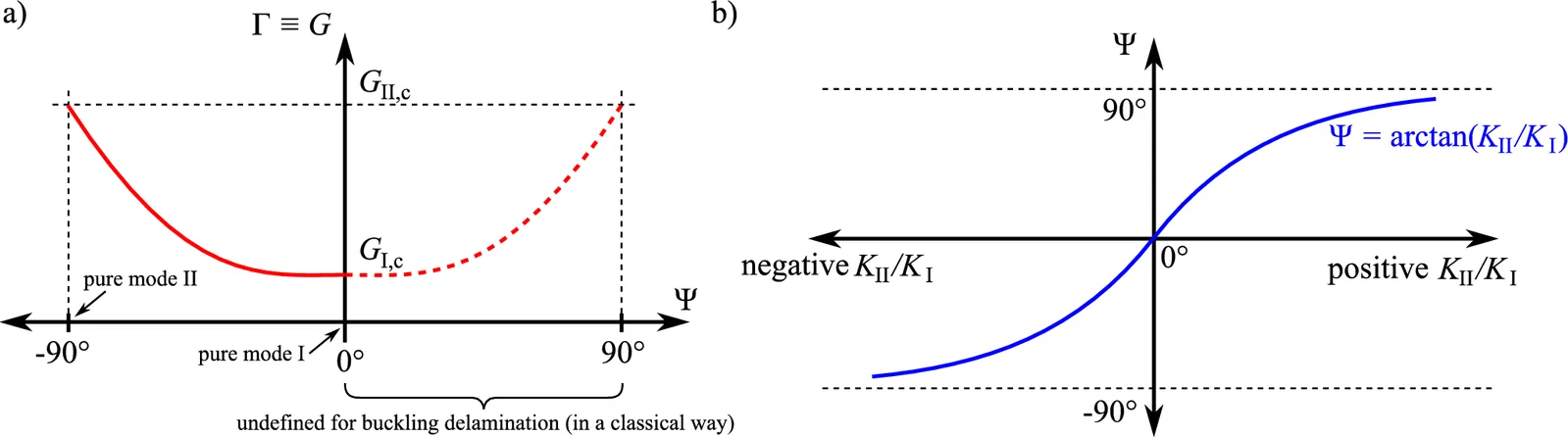
Schematic of the relationships between: (a) adhesion energy, Γ, and mode-mixity angle, Ψ, as seen by the classical approach, and (b) mode-mixity angle, Ψ, and the mode II to mode I loading ratio.

The classical approach sees the relationship between the adhesion energy, Γ, and mode mixity, Ψ, as depicted in Fig. 6a.^14,57–59^ The mode II adhesion energy (associated with *G*_II, c_) is always larger than mode I (*G*_I, c_) with a smooth transition and symmetrical behavior along the *y*-axis. As has been mentioned, the positive side of the graph in Fig. 6a is considered as “unidentified”, and any value falling into this sector should be deemed pure mode II loading with Ψ = − 90°. The reason behind this constriction is rooted in the positivity or negativity of the actual mode I and mode II loadings. As is visible from the schematic in Fig. 6b, the mode-mixity angle, Ψ, can be positive only if the *K*_II_ to *K*_I_ ratio is also positive, and negative Ψ can appear only with a negative *K*_II_ to *K*_I_ ratio. Due to the compressive stress in the thin film during the buckling–delamination, the shear direction will always correspond to negative mode II loading, therefore, negative *K*_II_ is always present. In combination with the tangential relationship between Ψ and the modes ratio, this leads to the conclusion that negative values of mode-mixity angle Ψ are associated only with positive *K*_I_, and positive mode-mixity angle Ψ only with negative *K*_I_. Based on this conclusion, the original works on buckling-induced delamination assumed that negative mode I loading (i.e., compression, interface crack is being closed) does not contribute to the delamination process and should be omitted. It has to be noted that this works only in the case of buckling-induced delamination with compressive residual stress in the film, which dictates the direction of mode II loading. For different delamination processes, the relationships described above do not apply.

While the discarding of the negative mode I loading makes sense in terms of the actual delamination processes (in a quasi-static crack propagation, without any fatigue processes), the effects of crack faces being pushed towards each other should not be omitted in general. This is also supported by the data presented in the “Results” section, showing interesting behavior, replicable through different material systems. Focusing on the negative part of graphs in Figs. 3, 4, and 5, two distinct characteristics can be found:

1. The pure mode II value of Γ_II_ is almost identical for both sides when Ψ =  ± 90°, counting only the results for the W-PMMA and Mo-Cu-PI material systems, since the W-SiO_2_ material system did not yield any positive mode-mixity angle; however, the two subsets yield the same mode II value, as is summarized in Table I.
2. Decreasing the mode-mixity angle from 90°, effectively increasing the contribution of negative mode I loading, shows a steep increase in apparent adhesion between the thin film and the substrate.

**Table I:** Summary of the pure mode I and II Γ values

Material system	Γ_I_(0°)/J m^−2^	Γ_II_(− 90°)/J m^−2^	Γ_II_(90°)/J m^−2^
W-SiO_2_, subset 1	–	–	0.72 ± 0.12
W-SiO_2_, subset 2	–	–	0.62 ± 0.06
W-PMMA	–	3.12 ± 0.16	3.19 ± 0.05
Mo-Cu-PI	0.0287 ± 0.0052	0.376 ± 0.026	0.381 ± 0.03

It should be noted that pure mode I adhesion energy could be calculated only for the Mo-Cu-PI material system with the fit of negative Ψ using Eq. 7, as presented in Table I. For other cases, there was not enough (or any) data points in the negative part, and the positive Ψ-region behavior is unknown for values approaching Ψ = 0°. For further research purposes, all the presented data points are made available through the Zenodo repository.^63^

### Impact on Pure Mode II Case (Ψ =  ± 90°)

The first characteristic is easy to explain, as, at both extreme cases when Ψ = ± 90°, only pure mode II loading is acting at the delamination crack front. There are no geometrical or loading differences between these cases; therefore, these have to yield the same material system-characteristic value. The implications of allowing for positive Ψ in this regard is a more accurate calculation of pure mode II adhesion energy and, subsequently, through the analytical model, the mode I interface toughness (*G*_I, c_). For instance, the pure mode II value for the Mo-Cu-PI material system could be estimated as 0.6 ± 0.3 J m^−2^ if all the positive Ψ data points would be considered pure mode II (a straightforward mean value from the whole dataset), almost doubling the estimated interface adhesion and with 50% standard deviation. A similar case would be the W-PMMA material system, leading to a pure mode II value of 3.6 ± 0.6 J m^−2^, overestimating the mode II adhesion by ~ 15% and increasing the standard deviation from 5% to 17%. Therefore, even without further analysis, plotting the results including the positive mode-mixity angles can significantly improve the adhesion measurements via buckling-induced delamination, and, with the use of only the real negative part of the graphs, could lead to more precise calculation of Γ_I_ (*G*_I,c_).

### Impact of Negative Mode I Loading when Ψ > 0°

Assuming no influence of the negative mode I loading on the delamination processes and the crack tip stress singularity, there is still some contribution of these phenomena on the cracking processes described in classical fracture mechanics. While standard fracture experiments designed to measure *G*_I, c_ are made to allow only for crack opening,^64–66^ which, in technical applications this is the most dangerous case, crack closure (negative mode I loading) can have a direct and indirect impact on the fracture processes. The direct impact is mostly related to fatigue loading where the cyclic plastic zone at the crack front affects the overall crack propagation even when it is caused by the compressive stresses, causing different results between positive and negative mode I loading, as well as being used for pre-cracking of the sample.^67–72^ However, the buckling-induced delamination is not generally a fatigue process; therefore, the indirect influence of negative mode I loading is more important in the present work. The stress field generated from the compressive loading indirectly affects the mode II component as well as crack faces pushing towards each other (when the crack is being closed by negative mode I loading). This will inevitably cause more interaction and friction between the delaminated surfaces, further increasing the apparent necessary energy for the actual shear delamination.^73–78^

Looking at the case of buckling-induced delamination with negative mode I loading, the crack closure will induce some additional friction and the apparent delamination energy will increase due to the energy dissipation on the friction, which is exactly what is visible in the present results. Higher mode I fraction is increasing the apparent adhesion, assuming sufficient shear component being affected by the friction.

**Fig. 7:**
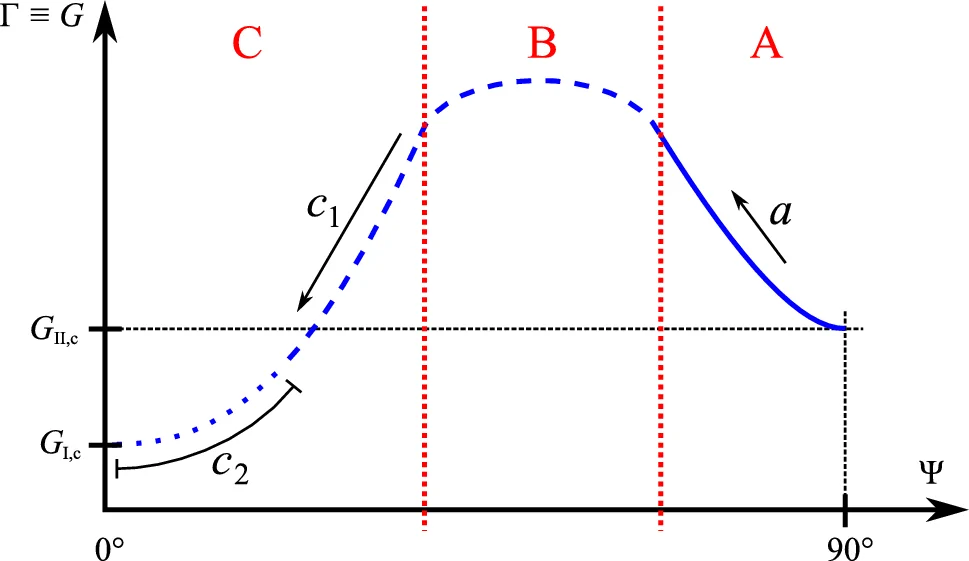
Newly proposed model of Γ–Ψ behavior during buckling-induced delamination for positive mode-mixity angles.

To accommodate the mentioned behavior and the crack surface friction, based on the measured and presented data, a new model of Γ–Ψ behavior is proposed (see Fig. 7), where the full range of positive mode-mixity angle Ψ is divided into three regions:

(A) Increase of negative mode I loading with high impact on shear delamination
  Even a small contribution of the crack surfaces pushing towards each other for high mode-mixity angles adds a frictional component due to a strong shear deformation of the crack front, with both loading modes works in conjunction, rapidly increasing the apparent adhesion energy along the arrow *a* in Fig. 7 with even a small decrease of mode-mixity angle from 90°.
  This is clearly visible on presented results in Figs. 2, 3, and 4.

(B) Competition between increasing friction and decreasing shear loading
  While the mode I component is still increasing in absolute value with mode-mixity angle going below the region A limit, the shearing through mode II loading decrease, effectively negating the outcome of the friction between the crack surfaces.
  A sign of plateau in results is visible in Fig. 4 for Ψ < 88°, while other investigated material systems have not yet reached the plateau, due to the different stress conditions, surfaces, and varying magnitudes of elastic mismatch between thin films and substrates, across the three tested configurations. Only a small hint of a starting plateau can be visible in Fig. 3 on the results of W-SiO_2_, subset 2; however, this cannot be confirmed quantitatively, only qualitatively from the number of data points being below the parabolic fit for Ψ < 65°.

(C) Decrease of crack surface friction influence on reduced shear loading
  While the contribution of negative mode I loading is even larger for smaller mode-mixity angles in region C, the contribution of mode II is small enough to not affect the overall apparent adhesion energy through the friction and decreases along the arrow *c*_1_ in Fig. 7.
  While there are no available results to support this part of the model, the existence of the competition region B suggests that the actual influence of the friction should be decreasing for lower mode-mixity angles and at some point it should vanish.
  Additionally, the pure mode I case for Ψ = 0° is set by the delamination behavior from the negative portion of the graph, and it is safe to expect the progression to be smooth from both sides of the Ψ-axis. However, since there are not enough data to support or disprove this assumption, we are aware of the fact that, due to a possibly small impact on delamination from the almost purely negative mode I loading, there might be a discontinuity in the graph presented in Fig. 7. Therefore, the portion marked as length *c*_2_ should be currently taken only as speculative and pending more research.

The negative mode I loading is observably impacting the apparent adhesion energy from the measurement, when a purely energetic approach is being used. It is true that, if the stress intensity approach would be taken, the negative mode I loading would have no impact on the formation of the crack tip singularity. From such a point of view, the original assumptions of only mode II loading acting at the crack front would be correct. However, as the purely energetic approach in the results shows, the negative mode I loading is influencing the energy balance through the crack surface friction, and has to be taken into account in the buckling-induced delamination technique, since the original approach is in fact based on the energy balance of the system.

It should be noted that the borders of each region A, B, and C are not set and are strongly dependent on the material system, including the interface roughness, the magnitude of the residual stress within the thin film, and the elastic mismatch between the film and the substrate. Therefore, the model presented in Fig. 7 is not fixed and more research has to be carried out to identify the actual contribution of the interface conditions and stress state of the thin film, to discover what is responsible for the negative mode I loading appearance and how the parameters affect the final outcome.

### Differences Between Material Systems

Comparing the different material systems, one can see that, although the general behavior for Ψ > 0° is the same, different material combinations do not show the same magnitude of the negative mode I loading effects and the distribution of regions A, B, and C from model presented in Fig. 7. This is best observed in a relative plot of the region A fits of all three material systems normalized to their respective mode II Γ values.

**Fig. 8:**
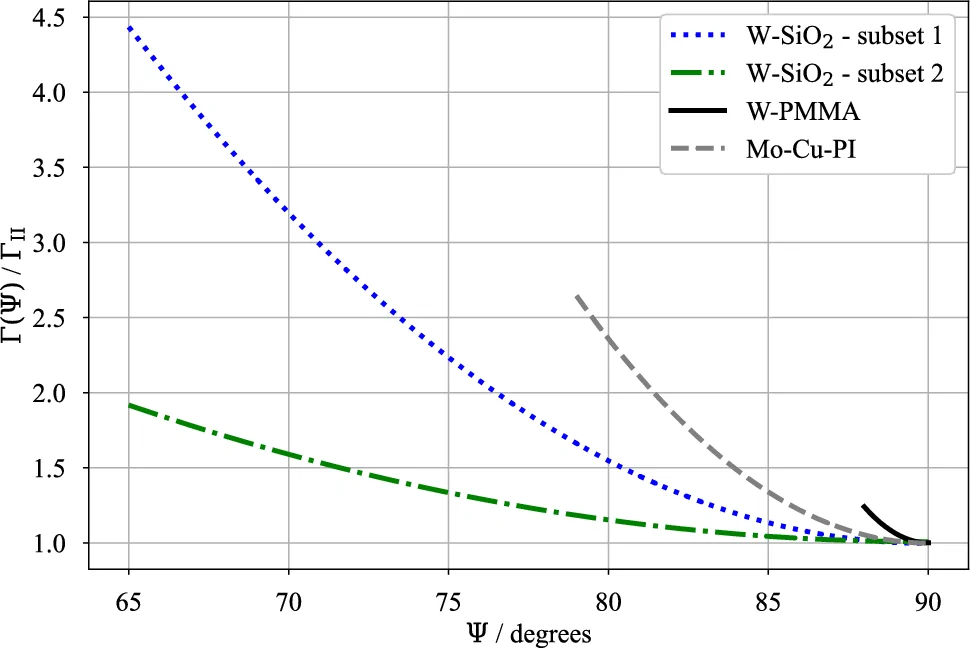
Region A fits for all the investigated material systems normalized to their respective mode II Γ values.

The first visible thing in the normalized plot in Fig. 8 is that the mode-mixity angle range is widely different. This is caused simply by the fact that the actual buckling (which could be observed) does not produce lower positive mode-mixity angles than the ones presented, as the buckling-induced delamination mostly favors the mode II loading.^14^ Therefore, with the exclusion of the W-PMMA material system, only region A was observed. For the W-PMMA material system only, small hints of plateau region B were observed. Therefore, for the investigated material systems, the buckles’ preferred loading regime was within region A of the proposed model (when negative mode I loading was present), and only a special combination of elastic mismatch, thin film thickness, and residual stresses in the W-PMMA material system led to different behavior.

Secondly, and most importantly, the speed and magnitude of the increase of the apparent adhesion energy increase is vastly different for different material systems. While the investigated results lead to a maximal increase of roughly 50% for the W-PMMA material system, the Mo-Cu-PI material system exhibits an increase to more than 2.5 times larger Γ than the pure mode-II value. The W-SiO_2_ material system is even more interesting. While the subset 1 reaches almost 4.5 times higher than its mode II value, the same material and interface in subset 2 reaches only 2 times the mode II value. One cause can be the different elastic mismatch between the thin film and the substrate materials, which differ significantly between each of the three material systems. However, that would not explain the differences between the two subsets for the W-SiO_2_ case. Here, the differences between the subsets in terms of the film thickness and residual stress in the films are interesting. When comparing the line-force acting on the thin films and calculated as a simple multiplication of the residual stress,* σ*, by the film thickness, *h*, the result for subset 1 is *F*_l_W-SiO2-01_ = 598.5 ± 89.0 N m^−1^, and for subset 2 is *F*_l_W-SiO2-02_ = 356.4 ± 34.2 N m^−1^. Even for a similar range of mode mixity, these two acting forces are largely different for each subset, resulting in different levels of negative mode I influence. The larger force for subset 1 pushes the thin film more into the substrate, leading to a larger effect of negative mode I loading through the friction. To fully complete the analysis, the line-force was also calculated for the other two material systems, resulting in *F*_l_W-PMMA_ = 3617 ± 636 N m^−1^ and *F*_l_Mo-Cu-PI_ = 222.6 ± 63.5 N m^−1^, counting only the buckles with Γ > 0°. While comparing the line-force values between different material systems omits the other effects, it can be safely observed that higher *F*_l_ values lead to a faster increase in Γ(Ψ) even for a small decrease of Ψ from 90°, as is most visible in the case of the W-PMMA system, and from comparing the two subsets of the W-SiO_2_ system. The Mo-Cu-PI material system not fitting this trend only underlines the other effects of the elastic mismatch on the overall deformation, and, thus, the level of the effect of negative mode I loading, since the substrate in this system is very soft and deformable, in comparison to the hard SiO_2_ and the soft, but rigid-enough, PMMA. Therefore, the stress state (or rather the loading state) of the thin film can be identified as one of the main influencing parameters on the effect of negative mode I loading on the apparent adhesion energy in the buckling-induced delamination. The second most likely influencing parameter is probably the interfacial roughness between the thin film and the substrate, However, there was no useful information on interface roughness available for the W-SiO_2_ and W-PMMA material systems. While the Mo-Cu-PI material system exhibited nanometer-scale smoothness of the PI surface, it could not be compared with the other material systems, and therefore is not discussed here. Additionally, the occurrence of plastic deformation within the thin film might influence the overall deformation and stress state of the buckle. As has been shown previously, contrary to the original pure elasticity assumption, buckles can exhibit a certain degree of plastic deformation.^34,35^ A similar thing can be said about the substrate, as the soft PMMA substrate in the presented W-PMMA case, as well as in the other material systems with more rigid (in terms of elastic modulus) thin films or soft interlayers, such as the W-Cu combination in Cordill et al.,^32^ can exhibit permanent deformation leading to forces acting against the crack opening. While the presented work cannot provide enough data to compare (for the same reason as for the surface roughness), the effects of permanent deformation within either the thin film or the substrate should be further investigated.

It has to be noted that the presented results and discussion do not offer an exact solution to the problem of negative mode I loading effects in buckling-induced delamination. This is not possible from only three material systems with vastly different stresses acting on the films with various thicknesses. However, since the current approach is to simply ignore the negative mode I loading influence, the presented results obtained from the new measurements as well as from older, but still viable, data offer a new approach which can, at worst, improve pure mode II adhesion evaluation precision, but, in general, opens a new field of view on the measured buckles by including the friction between the delamination crack surfaces. The model presented in Fig. 7 simply offers a scheme of how we envision the buckle to behave.

## Conclusion

This thorough analysis of the raw data from work carried out by research groups around Gerberich as well as analysis of the newly measured data presented in this work opens a new field of view on the positive mode-mixity angles during buckling-induced delamination. The results show that, in material systems with large elastic material property mismatches between the thin films and the substrate, as well as highly stressed thin films, buckles form under the Ψ > 0° condition, meaning negative mode I loading at the delamination crack front. This loading condition leads to crack surfaces to be pushed towards each other, causing significant friction and, thus, sudden increases in the measured apparent adhesion energy.

This work offers the first systematic study of this phenomenon with initial guidance as what might the allowing of negative mode I loading in analytical models bring into the field of buckling–delamination. Additional studies will have to be carried out on different material systems to fully develop such a model. However, the presented study shows the general behavior with satisfying proofs.

## Data Availability

The data that support the findings of this study are openly available in the Zenodo repository.^63^
